# Radioactive Iodine Therapy Decreases Recurrence in Thyroid Papillary Microcarcinoma

**DOI:** 10.5402/2012/816386

**Published:** 2012-03-07

**Authors:** Kimberly M. Creach, Barry A. Siegel, Brian Nussenbaum, Perry W. Grigsby

**Affiliations:** ^1^Department of Radiation Oncology, Washington University School of Medicine, 4921 Parkview Place, Mailstop no. 90-38-635, St. Louis, MO 63110, USA; ^2^Division of Nuclear Medicine, Mallinckrodt Institute of Radiology, Washington University School of Medicine, 660 S. Euclid Avenue, Campus Box 8223, St. Louis, MO 63110, USA; ^3^Department of Otolaryngology Head and Neck Surgery, Washington University School of Medicine, McMillan Hospital, 517 S. Euclid Avenue, St. Louis, MO 63110, USA; ^4^Division of Nuclear Medicine, Department of Radiation Oncology, The Alvin J. Siteman Cancer Center, Mallinckrodt Institute of Radiology, Washington University School of Medicine, 4921 Parkview Place, Mailstop #90-38-635, St. Louis, MO 63110, USA

## Abstract

*Background*. The most appropriate therapy for papillary microcarcinoma (PMC) is controversial. *Methods*. We reviewed the therapy and outcome of 407 patients with PMC. *Results*. Three hundred-eighty patients underwent total thyroidectomy, and 349 patients received I-131 therapy. The median followup was 5.3 years. Forty patients developed recurrent disease. On univariate analysis, development of disease recurrence was correlated with histological tumor size > 0.8 cm (*P* = 0.0104), age < 45 years (*P* = 0.043), and no I-131 therapy (*P* < 0.0001). On multivariate analysis, histological tumor size > 0.8 cm, positive lymph nodes, and no I-131 therapy were significant. The 5-year RFS for patients treated with I-131 was 95.0% versus 78.6% (*P* < 0.0001) for patients not treated with I-131. Patients with lymph node metastasis who did not receive I-131 had a 5-year RFS of 42.9% versus 93.2% (*P* < 0.0001) for patients who received I-131. *Conclusions*. Recommend I-131 remnant ablation for patients with PMC, particularly patients with lymph node metastasis.

## 1. Introduction

Papillary thyroid cancer is associated with an excellent prognosis, with cancer-specific survival of >90% [1, 2]. Tumor size has been established as prognostic factor [3–5]. As such, the World Health Organization defined tumors ≤1 cm in greatest dimension as a separate entity, papillary microcarcinoma (PMC) [6]. Given the overall excellent prognosis of PMC, there is controversy regarding the most appropriate treatment as one must weigh recurrence risk versus treatment toxicity. The appropriate extent of surgery and the utility of I-131 therapy in patients with PMC are unclear. Treatment recommendations, represented in the literature, range from observation alone to total thyroidectomy followed by I-131 therapy [7–10].

The purpose of this study is to identify patient and tumor prognostic factors for recurrent disease in patients with PMC, and to examine the impact of I-131 therapy for patients with PMC.

## 2. Methods and Materials

All patients with thyroid cancer who were referred to the Department of Radiation Oncology at Washington University were entered into a departmental database beginning in 1959. This data set was interrogated to identify patients older than 18 years with papillary microcarcinoma (≤1.0 cm). Four hundred-seven patients meeting these inclusion criteria were identified. Medical records were reviewed, and the following data were reviewed for this group: demographic information, extent of initial surgery, various histological parameters, I-131 treatment, and outcome. Lymph node status was determined from pathology reports and whole-body I-131 scintigraphy (WBS) results.

The majority of patients underwent total thyroidectomy (*n* = 380, 93.4%); 25 patients underwent lobectomy and 2 had surgery of unknown extent. Surgery was followed by I-131 therapy in 349 patients (85.7%). The reason for omitting I-131 in the remaining 14.3% of patients is unknown. No patients underwent >1 I-131 administration as initial therapy. The median administered activity of I-131 was 100 mCi (range 25–250 mCi). Three to 5 days after treatment, WBS was performed. Followup consisted of regularly scheduled physical examination, measurement of serum thyroglobulin (Tg) level (after the mid-1990s), and diagnostic WBS (performed with 5 mCi I-131).

The patient population consisted of 321 woman and 86 men; their median age at diagnosis was 45 years (range 18–80 years). The tumor histology was described as pure papillary in 317 patients, follicular variant in 87 patients, and tall cell variant in 4 patients. The median tumor size was 0.7 cm (range 0.1–1.0 cm). Additional histological features are depicted in Table 1. The patients were followed for a median of 5.3 years (range from 0.2 to 51 years).

Logistic regression analysis was performed to determine the correlation between various parameters and the development of recurrent disease. Multivariate logistic regression was done using Cox proportional hazards modeling. Recurrent disease was defined as the development of positive WBS on clinical report, positive FDG-PET on clinical report, positive pathology, or a detectable Tg level (>1 ng/mL), after a disease-free interval. The time to recurrence was defined as difference between the date of initial surgery and date of recurrence. Recurrence-free survival was based on the development of recurrent thyroid cancer. The Kaplan-Meier method was used to calculate survival rates.

## 3. Results

Of the 407 total patients, 40 (9.8%) developed recurrent disease at a median of 4.2 years (0.3–38 years). The location of first recurrence was as follows: 23 patients had disease recurrence in neck nodes, 10 in the thyroid bed only, 3 in multiple distant sites, 2 in the neck and lung, 1 in the lung only, and 1 by Tg elevation without a site of disease identified on imaging. Salvage therapy consisted of surgery in 5 patients, I-131 therapy in 17 patients, both surgery and I-131 therapies in 14 patients, and observation in 4 patients. Three (0.7%) patients died of thyroid cancer at a median of 38.6 years after diagnosis.

On univariate analysis, the development of disease recurrence was correlated with histological tumor size > 0.8 cm (*P* = 0.0104), age < 45 years (*P* = 0.043), and no I-131 therapy (*P* < 0.0001). We found no correlation between the development of recurrent disease and gender, histological subtype, positive lymph nodes on first WBS or pathology, vascular invasion, capsular invasion, soft tissue invasion, or positive surgical margins. On multivariate analysis, histological tumor size > 0.8 cm, positive lymph nodes, and no I-131 therapy were significant (Table 2). There was no threshold for tumor size below which patients did not experience recurrence; patients with recurrent disease had a median tumor size of 0.95 cm (0.1–1.0 cm).

**Figure 1:**
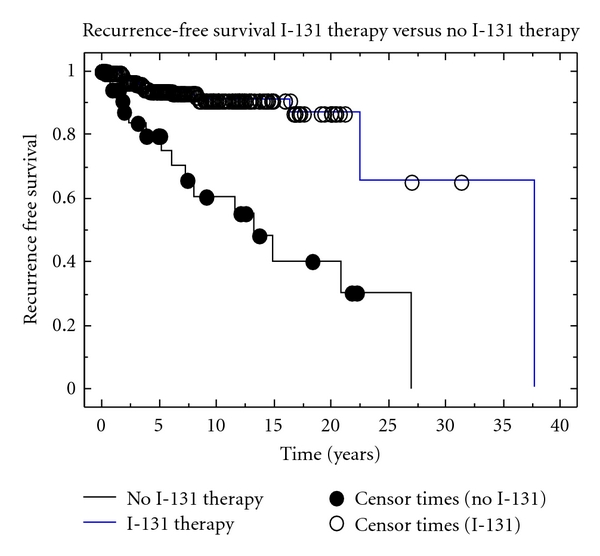


The 5-year RFS for patients treated with I-131 was 95.0% compared to 78.6% for patients not treated with I-131 (*P* < 0.0001) (Figure 1). I-131 therapy continued to be associated with improved RFS in the 231 patients with tumors ≤0.8 cm; the 5-year RFS were 96.3% and 92.6%, respectively, (*P* = 0.004) for such patients treated and not treated with I-131.

**Figure 2:**
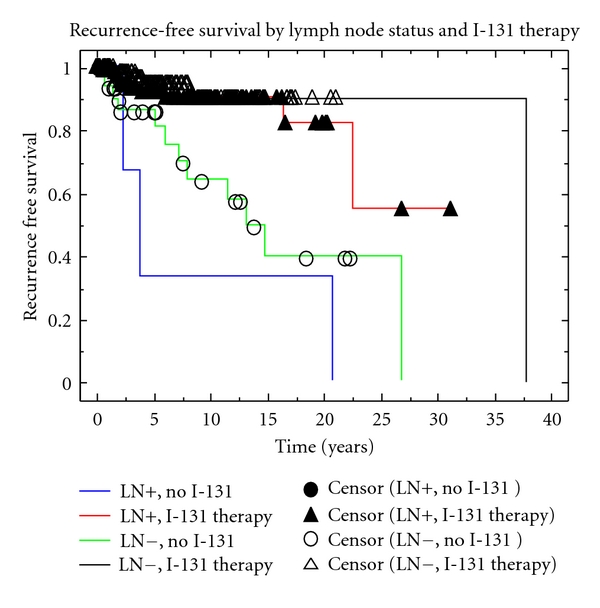


Patients with and without lymph node metastasis treated with I-131 had 5-year RFS of 93.2% and 96.2%, respectively (*P* = NS). Patients not treated with I-131 experienced a significantly worse 5-year RFS compared to those treated with I-131 regardless of lymph node status. Patients without lymph node metastasis who were not treated with I-131 had a 5-year RFS of 88.0% versus 96.2% (*P* < 0.0001), for patients treated with I-131. Patient with lymph node metastasis who did not receive I-131 had a 5-year RFS of 42.9% versus 93.2% (*P* < 0.0001) for patients who received I-131 (Figure 2).

Acute toxicity from I-131 was not recorded. Only one patient treated with I-131 had developed a chronic toxicity, lacrimal duct stenosis (0.3%).

## 4. Discussion

Patients with PMC have very low mortality [3, 11, 12]. However, 4–16% of patients with PMC develop recurrent disease with many of these patients developing distant metastasis [3, 8, 11–14]. As such, an appropriate balance between insufficient treatment, putting patients at risk for recurrence, and overly aggressive therapy, subjecting patients to undo toxicity, must be found for patients with PMC.

We were able to identify several treatment- and tumor-related prognostic factors in our cohort. However, our population represents patients referred for consideration of 1-131 therapy at a large academic medical center. As a result, our population may not be representative of the entire population with PMC but may be a higher-risk population. However, the RFS and cancer mortality for our patients are in line with those reported in other studies [3, 8, 11–14]. Further, our series is among the largest studied to date.

Lymph node status and tumor size were the only tumor-related independent risk factors for recurrence in our cohort. Many authors have advocated the use of tumor size to dictate treatment decisions Lo rt al. [15], Noguchi et al. [16], Roti et al. [17], Lin et al. [18]. Although we found tumor size >0.8 cm to be associated with recurrence, we were unable to identify a size threshold below which there was no risk of recurrence. Further, the prognostic value of lymph node status in patient with PMC has been well established [11, 13, 19], and lymph node status at presentation may be independent of tumor size in PMC [11, 20, 21]. In addition, the risk of positive lymph nodes at diagnosis for PMC has been reported to be 25–43.3% [8, 11–13]. Therefore, basing treatment decisions solely on tumor size seems inappropriate.

Age greater than 45 years at diagnosis is a poor prognostic factor in papillary thyroid cancer (AJCC staging manual 2009). However, age has not consistently been found to predict for outcome in patients with PMC [11, 20] suggesting that other risk factors, such as lymph node status, soft tissue invasion, and vascular invasion, are more important predictors of cancer biology than age in patients with small thyroid tumors [11–14, 20]. In our cohort, we found that age less than 45 years at diagnosis was a poor prognostic factor on univariate analysis. However, the younger patients in our cohort had larger tumors, more frequent lymph node metastasis, and were less likely to receive I-131 therapy (data not presented).

Multifocality has been reported in more than 30% of patients with PMC [3, 7, 11, 20]. Although multifocality was not predictive of outcome in our cohort, several authors have identified multifocal disease as a poor prognostic factor [11–14, 20]. Given the high rates of multifocality and the possible association with poor outcome, we advocate total thyroidectomy for both therapeutic and prognostic purposes.

Although there is a body of literature that advocates the use of I-131 therapy for patients with PMC (particularly those patients with poor histological features) (ATA guidelines) [7, 11, 12, 22, 23], few studies have been able to demonstrate a clinical benefit of I-131 therapy for patients with PMC [11]. In our cohort, I-131 treatment was correlated with improved RFS; patients undergoing I-131 therapy had a 5-year RFS of 96%. I-131 therapy also was associated with improved RFS in the subsets of our patients with tumors ≤0.8 cm and in node-negative patients. In addition to this apparent therapeutic benefit, remnant ablation with I-131 allows for increased sensitivity of thyroglobulin measurements for detection of recurrences (ATA). WBS after I-131 therapy also provides additional prognostic information by more frequent identification of lymph node and distant metastasis compared with pretreatment diagnostic I-131 scans [24, 25].

Although I-131 therapy is well tolerated, it is not without toxicity. Patients undergoing I-131 treatment experience both acute and chronic toxicity. Prior to treatment, patients are frequently profoundly hypothyroid. However, the availability of recombinant human thyroid stimulating hormone allows patients to be treated with I-131 without hypothyroidism associated with thyroid hormone withdrawal [26, 27]. After treatment with I-131, patients rarely experience headache, fatigue, nausea, and vomiting acutely [28]. More commonly, patients develop acute sialoadenitis [29]. Typically, acute toxicity resolves rapidly. Patients also can develop chronic toxicity from I-131, such as xerostomia and subsequent dental caries [30], lacrimal duct dysfunction [31], chronic sialoadenitis [32], and rarely second malignancy [33]. I-131 toxicity is dose dependent; patients receiving higher activities of I-131 (>150 mCi) experience increased toxicity [34]. However, despite these toxicities, the long-term quality of life of patients treated for differentiated thyroid cancer has been demonstrated to be similar to that of the normal population [35].

As noted above, 4–16% of patients with PMC develop recurrent disease [3, 8, 11–14]. Cancer recurrence not only negatively affects physical health, but also impacts mood, stress, and overall quality of life [36–38]. Furthermore, fear of recurrence is associated with poor quality of life for cancer patients and their families [39, 40]. While not specifically studied in thyroid cancer, treatment decisions have been shown to impact fear of cancer recurrence [39, 41, 42]. Further study is needed to determine if more aggressive therapy for PMC, such as I-131 treatment, would allow for improved quality of life by decreasing recurrence or fear of recurrence.

## 5. Conclusions

Although patients with PMC have excellent overall survival, a subset of patients will develop recurrent disease. We were able to identify clinical characteristics that predicted for recurrent disease: greater tumor size, positive lymph nodes, and lack of I-131 therapy. We thus recommend that patients with PMC, particularly those with involved lymph nodes, be treated with thyroidectomy followed by thyroid remnant ablation with I-131.

## Figures and Tables

**Table 1:** Characteristics of patient population (*N* = 407).

Characteristic	*N (%)*
Age: median (range)	45 years (18–80)
Gender	
Male	86 (21.1)
Female	321 (78.9)
Extent of surgery	
Lobectomy	27 (6.6)
Total thyroidectomy	380 (93.3)
Multifocal tumor	188 (46.2)
Capsular invasion	126 (31.0)
Vascular invasion	14 (3.4)
Soft tissue invasion	68 (16.7)
Positive surgical margin	73 (17.9)
positive lymph node	153 (37.6)
Tumor size > 0.8 cm	132 (32.4)

**Table 2:** Logistic regression analysis for correlation of various risk factors with development of recurrent disease.

Characteristic	Number with recurrence (%)	Univariate *P* value	Relative risk (95% CI)	Multivariate *P* value
Age				
≥45 years	10/207 (4.8)			
<45 years	30/200 (15.0)	0.042		NS
Gender				
Male	7/86 (8.1)			
Female	33/321 (10.3)	0.248		NS
Multifocal tumor				
Yes	20/188 (10.6)	0.462		NS
No	20/219 (9.1)			
Capsular invasion				
Yes	15/126 (11.9)	0.389		NS
No	25/281 (8.9)			
Vascular Invasion				
Yes	3/14 (21.4)	0.823		NS
No	37/393 (9.4)			
Soft tissue invasion				
Yes	8/68 (11.8)	0.937		NS
No	32/339 (9.4)			
Positive surgical margin				
Yes	8/73 (11.0)	0.654		NS
No	32/334 (9.6)			
Positive lymph nodes				
Yes	19/153 (12.4)	0.4266	2.3 (1.2–4.6)	0.0150
No	21/252 (8.3)			
Tumor size				
>0.8 cm	25/132 (18.9)	0.0104	2.1 (1.1–4.1)	0.0250
≤0.8 cm	15/275 (5.5)			
I-131 therapy				
No	20/51 (39.2)	<0.0001	8.6 (4.4–17.1)	<0.0001
Yes	20/349 (5.7)			

## References

[1] Mazzaferri EL (2000). Long-term outcome of patients with differentiated thyroid carcinoma: effect of therapy. *Endocrine Practice*.

[2] Palme CE, Waseem Z, Raza SN, Eski S, Walfish P, Freeman JL (2004). Management and outcome of recurrent well-differentiated thyroid carcinoma. *Archives of Otolaryngology—Head and Neck Surgery*.

[3] Giordano D, Gradoni P, Oretti G, Molina E, Ferri T (2010). Treatment and prognostic factors of papillary thyroid microcarcinoma. *Clinical Otolaryngology*.

[4] Giles Senyurek Y, Tunca F, Boztepe H, Alagöl F, Terzioglu T, Tezelman S (2009). The long term outcome of papillary thyroid carcinoma patients without primary central lymph node dissection: expected improvement of routine dissection. *Surgery*.

[5] Baek SK, Jung KY, Kang SM (2010). Clinical risk factors associated with cervical lymph node recurrence in papillary thyroid carcinoma. *Thyroid*.

[6] Hedinger C, Williams ED, Sobin LH (1989). The WHO histological classification of thyroid tumors: a commentary on the second edition. *Cancer*.

[7] Sugitani I, Toda K, Yamada K, Yamamoto N, Ikenaga M, Fujimoto Y (2010). Three distinctly different kinds of papillary thyroid microcarcinoma should be recognized: our treatment strategies and outcomes. *World Journal of Surgery*.

[8] Arora N, Turbendian HK, Kato MA, Moo TA, Zarnegar R, Fahey TJ (2009). Papillary thyroid carcinoma and microcarcinoma: is there a need to distinguish the two?. *Thyroid*.

[9] Cappelli C, Castellano M, Braga M (2007). Aggressiveness and outcome of papillary thyroid carcinoma (PTC) versus microcarcinoma (PMC): a mono-institutional experience. *Journal of Surgical Oncology*.

[10] Cooper DS, Doherty GM, Haugen BR (2009). Revised American thyroid association management guidelines for patients with thyroid nodules and differentiated thyroid cancer. *Thyroid*.

[11] Chow SM, Law SCK, Chan JKC, Au SK, Yau S, Lau WH (2003). Papillary microcarcinoma of the thyroid-prognostic significance of lymph node metastasis and multifocality. *Cancer*.

[12] Mercante G, Frasoldati A, Pedroni C (2009). Prognostic factors affecting neck lymph node recurrence and distant metastasis in papillary microcarcinoma of the thyroid: results of a study in 445 patients. *Thyroid*.

[13] Hay ID, Hutchinson ME, Gonzalez-Losada T (2008). Papillary thyroid microcarcinoma: a study of 900 cases observed in a 60-year period. *Surgery*.

[14] Ross DS, Litofsky D, Ain KB (2009). Recurrence after treatment of micropapillary thyroid cancer. *Thyroid*.

[15] Lo CY, Chan WF, Lang BHH, Lam KY, Wan KY (2006). Papillary microcarcinoma: is there any difference between clinically overt and occult tumors?. *World Journal of Surgery*.

[16] Noguchi S, Yamashita H, Uchino S, Watanabe S (2008). Papillary microcarcinoma. *World Journal of Surgery*.

[17] Roti E, Rossi R, Trasforini G (2006). Clinical and histological characteristics of papillary thyroid microcarcinoma: results of a retrospective study in 243 patients. *Journal of Clinical Endocrinology and Metabolism*.

[18] Lin KD, Lin JD, Huang MJ (1997). Clinical presentations and predictive variables of thyroid microcarcinoma with distant metastasis. *International Surgery*.

[19] Fiorotto ML, Klish WJ (1991). Total body electrical conductivity measurements in the neonate. *Clinics in Perinatology*.

[20] Pisanu A, Reccia I, Nardello O, Uccheddu A (2009). Risk factors for nodal metastasis and recurrence among patients with papillary thyroid microcarcinoma: differences in clinical relevance between nonincidental and incidental tumors. *World Journal of Surgery*.

[21] Grigsby PW, Reddy RM, Moley JF, Hall BL (2006). Contralateral papillary thyroid cancer at completion thyroidectomy has no impact on recurrence or survival after radioiodine treatment. *Surgery*.

[22] Pelizzo MR, Boschin IM, Toniato A (2006). Papillary thyroid microcarcinoma (PTMC): prognostic factors, management and outcome in 403 patients. *European Journal of Surgical Oncology*.

[23] Küçük NÖ, Tari P, Tokmak E, Aras G (2007). Treatment for microcarcinoma of the thyroid—clinical experience. *Clinical Nuclear Medicine*.

[24] Fatourechi V, Hay ID, Mullan BP (2000). Are posttherapy radioiodine scans informative and do they influence subsequent therapy of patients with differentiated thyroid cancer?. *Thyroid*.

[25] Sherman SI, Tielens ET, Sostre S, Wharam MD, Ladenson PW (1994). Clinical utility of posttreatment radioiodine scans in the management of patients with thyroid carcinoma. *Journal of Clinical Endocrinology and Metabolism*.

[26] Dueren C, Dietlein M, Luster M (2010). The use of thyrogen® in the treatment of differentiated thyroid carcinoma: an intraindividual comparison of clinical effects and implications of daily life. *Experimental and Clinical Endocrinology and Diabetes*.

[27] Robbins RJ, Driedger A, Magner J (2006). Recombinant human thyrotropin-assisted radioiodine therapy for patients with metastatic thyroid cancer who could not elevate endogenous thyrotropin or be withdrawn from thyroxine. *Thyroid*.

[28] Perez CA, Brady LW, Halperin EC (2004). *Principles and Practice of Radiation Oncology*.

[29] Nakada K, Ishibashi T, Takei T (2005). Does lemon candy decrease salivary gland damage after radioiodine therapy for thyroid cancer?. *Journal of Nuclear Medicine*.

[30] Walter MA, Turtschi CP, Schindler C, Minnig P, Müller-Brand J, Müller B (2007). The dental safety profile of high-dose radioiodine therapy for thyroid cancer: long-term results of a longitudinal cohort study. *Journal of Nuclear Medicine*.

[31] Kloos RT, Duvuuri V, Jhiang SM, Cahill KV, Foster JA, Burns JA (2002). Nasolacrimal drainage system obstruction from radioactive iodine therapy for thyroid carcinoma. *Journal of Clinical Endocrinology and Metabolism*.

[32] Bomeli SR, Schaitkin B, Carrau RL, Walvekar RR (2009). Interventional sialendoscopy for treatment of radioiodine-induced sialadenitis. *Laryngoscope*.

[33] Sawka AM, Thabane L, Parlea L (2009). Second primary malignancy risk after radioactive iodine treatment for thyroid cancer: a systematic review and meta-analysis. *Thyroid*.

[34] Almeida J, Vartanian JG, Kowalski LP (2009). Clinical predictors of quality of life in patients with initial differentiated thyroid cancers. *Archives of Otolaryngology—Head and Neck Surgery*.

[35] Malterling RR, Andersson RE, Falkmer S, Falkmer U, Niléhn E, Jrhult J (2010). Differentiated thyroid cancer in a Swedish county long-term results and quality of life. *Acta Oncologica*.

[36] Yang HC, Thornton LM, Shapiro CL, Andersen BL (2008). Surviving recurrence: psychological and quality-of-life recovery. *Cancer*.

[37] Andersen BL, Shapiro CL, Farrar WB, Crespin T, Welis-DiGregorio S (2005). Psychological responses to cancer recurrence: a controlled prospective study. *Cancer*.

[38] Siddiqi A, Given CW, Given B, Sikorskii A (2009). Quality of life among patients with primary, metastatic and recurrent cancer. *European Journal of Cancer Care*.

[39] Rosmolen WD, Boer KR, De Leeuw RJR (2010). Quality of life and fear of cancer recurrence after endoscopic and surgical treatment for early neoplasia in Barretts esophagus. *Endoscopy*.

[40] Hodges LJ, Humphris GM (2009). Fear of recurrence and psychological distress in head and neck cancer patients and their carers. *Psycho-Oncology*.

[41] Härtl K, Janni W, Kästner R (2003). Impact of medical and demographic factors on long-term quality of life image of breast cancer patients. *Annals of Oncology*.

[42] Mehta SS, Lubeck DP, Pasta DJ, Litwin MS (2003). Fear of cancer recurrence in patients undergoing definitive treatment for prostate cancer: results from capsure. *Journal of Urology*.

